# Five-year trends in epidemiology and prevention of mother-to-child HIV transmission, St. Petersburg, Russia: results from perinatal HIV surveillance

**DOI:** 10.1186/1471-2334-11-292

**Published:** 2011-10-27

**Authors:** Dmitry M Kissin, Michele G Mandel, Natalia Akatova, Nikolay A Belyakov, Aza G Rakhmanova, Evgeny E Voronin, Galina V Volkova, Alexey A Yakovlev, Denise J Jamieson, Charles Vitek, Joanna Robinson, William C Miller, Susan Hillis

**Affiliations:** 1 Centers for Disease Control and Prevention, 4770 Buford Highway NE, MS K-34, Atlanta, Georgia 30341, USA; 2 City AIDS Center, 12 Bumazgnaya Street, St. Petersburg 198020, Russia; 3 City Health Committee, 12 Bumazgnaya Street, St. Petersburg 198020, Russia; 4 Republican Hospital of Infectious Diseases Clinical AIDS Center, January 9th Prospect, Ust Izgora 196645, Russia; 5 Botkin Hospital of Infectious Diseases, 3 Mirgorodskaya Street, St. Petersburg 191167, Russia; 6 Centers for Disease Control and Prevention, 19 Nizhny Val, Kiev 04071, Ukraine; 7 Elizabeth Glaser Pediatric AIDS Foundation, 11150 Santa Monica Boulevard, Suite 1050, Los Angeles, California 90025, USA; 8 University of North Carolina at Chapel Hill, 2105F McGavran-Greenberg Hall, Chapel Hill, North Carolina 27599, USA

## Abstract

**Background:**

The HIV epidemic in Russia has increasingly involved reproductive-aged women, which may increase perinatal HIV transmission.

**Methods:**

Standard HIV case-reporting and enhanced perinatal HIV surveillance systems were used for prospective assessment of HIV-infected women giving birth in St. Petersburg, Russia, during 2004-2008. Trends in social, perinatal, and clinical factors influencing mother-to-child HIV transmission stratified by history of injection drug use, and rates of perinatal HIV transmission were assessed using two-sided χ^2 ^or Cochran-Armitage tests.

**Results:**

Among HIV-infected women who gave birth, the proportion of women who self-reported ever using injection drugs (IDUs) decreased from 62% in 2004 to 41% in 2008 (*P *< 0.0001). Programmatic improvements led to increased uptake of the following clinical services from 2004 to 2008 (all *P *< 0.01): initiation of antiretroviral prophylaxis at ≤28 weeks gestation (IDUs 44%-54%, non-IDUs 45%-72%), monitoring of immunologic (IDUs 48%-64%, non-IDUs 58%-80%) and virologic status (IDUs 8%-58%, non-IDUs 10%-75%), dual/triple antiretroviral prophylaxis (IDUs 9%-44%, non-IDUs 14%-59%). After initial increase from 5.3% (95% confidence interval [CI] 3.5%-7.8%) in 2004 to 8.5% (CI 6.1%-11.7%) in 2005 (*P *< 0.05), perinatal HIV transmission decreased to 5.3% (CI 3.4%-8.3%) in 2006, and 3.2% (CI 1.7%-5.8%) in 2007 (*P *for trend <0.05). However, the proportion of women without prenatal care and without HIV testing before labor and delivery remained unchanged.

**Conclusions:**

Reduced proportion of IDUs and improved clinical services among HIV-infected women giving birth were accompanied by decreased perinatal HIV transmission, which can be further reduced by increasing outreach and HIV testing of women before and during pregnancy.

## Background

The HIV epidemic in Russia, historically concentrated among male injection drug users, continues to grow [1]. Russia has one of the world's highest prevalences of injection drug use and one of the world's largest numbers of injection drug users. The Reference Group to the United Nations on HIV and Injecting Drug Use estimates HIV seroprevalence to be 37.2% among this group [2]. Although injection drug use remains the driving force of the epidemic in Russia, there is growing evidence of its spread outside traditional risk groups to the general population [3,4]. The bridging of HIV to individuals who never injected drugs primarily occurs through unprotected sexual contacts with HIV-infected injection drug users, most of whom are young and sexually active [4,5]. The increase in sexual transmission of HIV disproportionately affects women, whose proportion among newly registered HIV cases in Russia increased from 13.0% in 1995 to 42.0% in 2008 [6].

In the absence of the full range of reproductive health services for HIV-infected women, the growing presence of women in the HIV epidemic may increase the number of infants exposed to HIV. Although prevention of mother-to-child HIV transmission is among the highest priorities in Russia, current transmission rates are still higher than observed in high-income countries, where elimination of perinatal HIV infection is a feasible goal [7,8]. The widespread prophylactic use of highly active antiretroviral therapy throughout pregnancy, one of the mainstays of preventing perinatal HIV transmission in North America and Western Europe, is underutilized in Russia [9]. Many barriers to effective perinatal prevention relate to either behavioral characteristics of HIV-infected women themselves (e.g., lack of family planning or prenatal care) or to the quantity and quality of clinical services provided to these women and their infants during pregnancy, labor and delivery, and the postpartum period (e.g., late or inadequate antiretroviral prophylaxis, lack of HIV disease progression monitoring) [10]. It is unclear whether trends in these behavioral and clinical barriers differ among those HIV-infected women who used injection drugs, compared with those who did not.

St. Petersburg, the second largest Russian city, has the highest number of people living with HIV and one of the highest number of deliveries by HIV-infected women in Russia [6]. Although the standard HIV case-reporting system is useful for measuring trends over time, it likely underestimates the true number of HIV-infected individuals because of limited outreach to high-risk groups and exclusion of HIV-infected inhabitants who are not *official *residents of the city. To supplement the standard HIV surveillance system with data that would inform prevention programs, an enhanced perinatal HIV surveillance system was established in St. Petersburg to monitor uptake of prevention activities and rates of perinatal HIV transmission [10]. The objectives of our analyses were to explore five-year trends in factors influencing perinatal HIV transmission. By using the standard HIV case-reporting system, we assessed trends in HIV seroprevalence among all women giving birth and trends in birth rates among HIV-infected women. Furthermore, we used enhanced perinatal HIV surveillance among HIV-infected women giving birth to explore overall trends in proportion of women with history of injection drug use before and during pregnancy; to evaluate trends in social, perinatal, and clinical factors influencing mother-to-child HIV transmission among women with and without history of injection drug use; and to assess whether these changes were accompanied by concurrent reductions in perinatal HIV transmission rates.

## Methods

### Study population and data collection

*The standard HIV case-reporting system *collects annual data on all HIV cases, including age and gender. Inclusion in the standard HIV surveillance requires a thorough and lengthy clinical evaluation at the City AIDS Center, which provides state-subsidized services for HIV-infected persons, including confirmatory testing, virologic infant diagnosis, antiretroviral treatment, care and support. Therefore, the number of HIV cases in the general population is severely underestimated [3]. At the same time, data on HIV among women giving birth are quite accurate because nearly all women in St. Petersburg and Russia deliver in medical facilities, and there has been high coverage with antenatal and natal HIV screening, although results were frequently not accessible to guide prevention decisions. In April 2004, the supplemental *enhanced perinatal HIV surveillance *was established in maternity hospitals dedicated to providing care for HIV-infected women or women with no or incomplete HIV testing during pregnancy [10]. Data were collected for all women who were HIV-infected at the time of delivery in these hospitals, including basic demographics, prenatal care, birth history, maternal HIV testing, antiretroviral therapy and/or prophylaxis, utilization of HIV-related clinical services, substance use, and infant HIV serostatus. This enhanced surveillance covered >90% of HIV-infected women giving birth in St. Petersburg (<10% of HIV-infected women deliver at one of the 14 low-risk maternity hospitals that provide delivery services primarily for women who have no serious infectious diseases).

### Variables and definitions

Data collected by using standard HIV surveillance allowed calculation of the following: (a) *HIV seroprevalence among women giving birth *based on the proportion of HIV-infected women among all women giving birth in St. Petersburg (by using annual reporting to the St. Petersburg City Health Committee), and (b) *birth rate among HIV-infected women *based on the proportion of HIV-infected women who gave birth in a given year among all HIV-infected women *registered *at the City AIDS Center for that year.

The enhanced perinatal HIV surveillance system used medical record abstraction to provide further data about a broader set of indicators for HIV-infected women giving birth. Information on the *history of injection drug use *before and during pregnancy, *maternal age*, *marital status*, *city residence*, *previous livebirths*, and *intendedness of pregnancy *was self-reported either at admission to the maternity hospital or during intrapartum hospitalization (information on maternal age and city residence is usually verified at admission, if identification is available). For the purposes of the study, injection drug users (IDUs) were defined as women who reported using injection drugs at any point in time, even if they were not current users. The *gestational age *was assessed at admission to the hospital by the attending obstetrician; p*reterm delivery *was defined as delivery at <37 weeks gestation. *Elective cesarean delivery *was defined as planned cesarean delivery performed on the basis of an obstetrical or medical indication before labor; emergency cesarean delivery was defined as cesarean delivery performed during labor.

The enhanced perinatal HIV surveillance also included information about laboratory tests for HIV-infected women. The Russian Ministry of Health recommends HIV testing twice during pregnancy-at the first prenatal care visit, and again after 34 weeks of gestation [11]. All women who were admitted for delivery and did not have a negative HIV test after 34 weeks of gestation (except for women who already had documented HIV infection) were tested with rapid HIV test (Determine, Abbott Laboratories, Abbott Park, IL) [10] and included in the current analysis if rapid test was positive (*HIV diagnosis at labor and delivery*). In addition, pregnant women are tested routinely for *hepatitis C virus *(HCV) by using anti-HCV enzyme immunoassay and RecombiBest anti-HCV polymerase chain reaction (PCR) test (Vector-Best, Novosibirsk, Russia) or COBAS Amplicor HCV Monitor test (Roche-Diagnostics, Germany) [12]. Hepatitis C virus is primarily transmitted through exposure to infected blood and has been used by some researchers as a biomarker for injection drug use [13]. It is recommended that all HIV-infected women have *immunologic and virologic monitoring *during pregnancy, starting from the first prenatal care visit [11]. *CD4 count *was assessed by using FACSCalibur (BD Biosciencies, San Jose, CA), and *viral load *was assessed by using Abbot RealTime HIV-1 (Abbott Laboratories, Abbott Park, IL) tests. The values of the last available CD4 and viral load test during pregnancy were used in the current analysis.

In Russia, it is recommended that HIV-infected pregnant women initiate *antiretroviral prophylaxis *at 22 weeks of gestation or continue antiretroviral treatment if they are already on therapy for their own HIV disease [11,14]. Acceptable antenatal prophylactic regimens include AZT monotherapy, dual therapy typically with AZT and lamivudine (3TC), or triple combination therapy typically with AZT, 3TC, and lopinavir/ritonavir (AZT monotherapy and AZT + 3TC dual therapy are acceptable options for prophylaxis when maternal viral load <1,000 copies/mL). In addition, intravenous AZT administered to the mother at the onset of labor followed by 4 weeks of AZT syrup for the infant are recommended. During earlier years (2004-2006), most women who received a dual or triple combination regimen needed ARV treatment for their own health, whereas most women who received AZT monotherapy for perinatal prophylaxis did not need ARV treatment for their own health. The infant's HIV infection status was determined by the case definition for surveillance used by U.S. Centers for Disease Control and Prevention (CDC) for perinatally exposed non-breastfeeding infants [15]: a) definitively HIV-infected (two or more positive PCR tests using separate blood specimens); b) presumptively HIV-infected (one positive PCR and no subsequent negative HIV tests); c) definitively HIV-uninfected (two or more negative PCR tests from separate specimens obtained at ≥1 month and ≥4 months of age and no other laboratory evidence of HIV infection); d) presumptively HIV-uninfected (two negative PCR tests, both at age ≥2 weeks and at least one test at age ≥4 weeks and no subsequent positive PCR tests; one negative PCR test performed at ≥8 weeks of age and no subsequent positive PCR tests; or one positive PCR test followed by at least two negative PCR tests with one test at age ≥8 weeks and no subsequent positive results). *Perinatal HIV transmission *was calculated as the proportion of infants who were definitively or presumptively HIV-infected among those who were born alive to HIV-infected women and had determined HIV status as of September 2008. Although information about breastfeeding after the discharge from the hospital was not available, local experts believe that most HIV-exposed infants received replacement feeding. Therefore, HIV infection in infants almost always represents perinatal HIV transmission. A newborn infant was considered *abandoned *if the paperwork on relinquishment of parental rights was initiated by the mother before discharge from the maternity hospital or if the mother left the hospital without the newborn.

### Statistical analysis

Data using standard HIV surveillance were calculated for each calendar year from 2004 to 2008. Data from the enhanced HIV surveillance were analyzed for each of the five one-year data collection cycles starting from April 13, 2004. For the purposes of this manuscript, we presented each year of data as the year when the data collection cycle began (e.g., the data collection cycle that started on April 13, 2004 and ended on April 12, 2005 was labeled as *2004 *and the data collection cycle that started on April 13, 2005 and ended on April 12, 2006 was labeled as *2005*. SAS version 9.2 (SAS Institute Inc., Cary, NC) was used for all analyses.

The trend analyses of dichotomous variables were conducted by using the two-sided χ^2 ^test; trend analyses of polychotomous (three or higher level) variables were conducted by using the Cochran-Armitage two-sided test. The Kruskal-Wallis and the Wilcoxon rank-sum tests were used to compare median CD4-cell counts and median viral load values.

### Ethical approvals

The project was reviewed for human subject concerns by the CDC and the St. Petersburg City Health Committee and was determined to be public health practice, not research. Therefore, review was not needed by an institutional review board.

## Results

**Figure 1:**
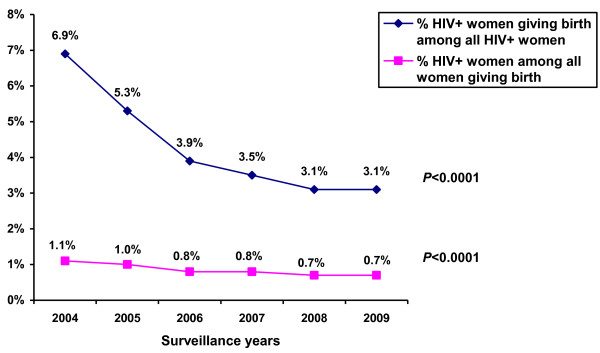
**HIV seroprevalence among women giving birth and birth rate among HIV-infected women**. Proportion of HIV-infected women giving birth among all HIV-infected women and proportion of HIV-infected women among all women giving birth, St. Petersburg, Russia, 2004-2009, standard HIV surveillance.

Analyses of the standard HIV surveillance revealed that although the overall number of deliveries in St. Petersburg during 2004-2009 increased, HIV seroprevalence of women giving birth decreased as follows: 1.1% (466/42,846) in 2004; 1.0% (415/41,471) in 2005; 0.8% (346/41,815) in 2006; 0.8% (355/46,239) in 2007; 0.7% (357/49,978) in 2008; and 0.7% (391/53,798) in 2009 (*P *< 0.0001). Although the number of identified HIV-infected women in the city increased, the birth rate among HIV-infected women decreased: 6.9% (466/6,763) in 2004, 5.3% (415/7,849) in 2005, 3.9% (346/8,976) in 2006, 3.5% (355/10,125) in 2007, 3.1% (357/11,405) in 2008, and 3.1% (391/12,695) in 2009 (*P *< 0.0001, Figure 1).

**Figure 2:**
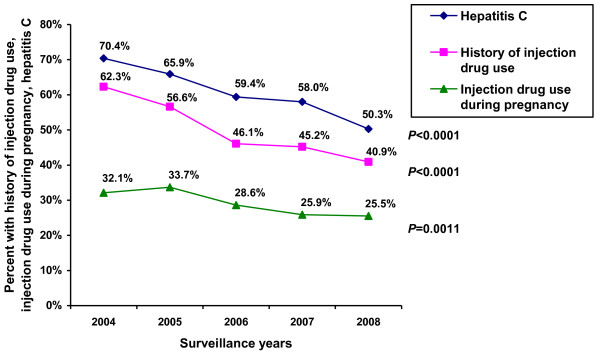
**History of injection drug use and prevalence of hepatitis C among HIV-infected women giving birth**. History of injection drug use, history of injection drug use during pregnancy, and prevalence of hepatitis C among HIV-infected women giving birth, St. Petersburg, Russia, 2004-2008, enhanced perinatal HIV surveillance.

According to the enhanced perinatal HIV surveillance system, the number and proportion of IDUs among HIV-infected women giving birth decreased from 62.3% in 2004 to 40.9% in 2008; similarly, the proportion of women coinfected with HCV decreased from 70.4% to 50.3% (both *P *< 0.0001). Although the decreasing proportion of IDUs was accompanied by a smaller proportion of women who reported injection drug use during pregnancy among *all *HIV-infected women (32.1%-25.5%, *P *< 0.005, Figure 2), the proportion of IDUs reporting injection drug use during pregnancy increased (51.6%-62.3%, *P *< 0.05, Table 1).

**Table 1:** Demographic, maternal and infant characteristics of HIV-infected women giving birth, by history of injection drug  use and surveillance year, St. Petersburg, Russia, 2004−2008, enhanced perinatal HIV surveillance.

	No history of injection drug use						History of injection drug use					
	**2004** **N = 209**^1^ **% (n) **	**2005** **N = 233**^1^ **% (n) **	**2006** **N = 246**^1^ **% (n) **	**2007** **N = 269**^1^ **% (n)**	**2008** **N = 264**^1^ **% (n)**	***P*-value**^2^	**2004** **N = 345**^1^ **% (n) **	**2005** **N = 304**^1^ **% (n) **	**2006** **N = 210**^1^ **% (n) **	**2007** **N = 222**^1^ **% (n) **	**2008** **N = 183**^1^ **% (n) **	***P*-value**^2^

**Maternal demographic characteristics and reproductive history**												
**Age**												.0007
<21 years	34.0 (71)	27.2 (63)	20.7 (51)	13.8 (37)	9.5 (25)	<.0001	13.0 (45)	11.5 (35)	6.2 (13)	6.8 (15)	6.0 (11)	
21-30 years	63.2 (132)	66.4 (154)	69.1 (170)	75.1 (202)	73.1 (193)	0.0026	84.1 (290)	80.3 (244)	84.8 (178)	82.0 (182)	80.9 (148)	0.5529
>30 years	2.9 (6)	6.5 (15)	10.2 (25)	11.2 (30)	17.4 (46)	<.0001	2.9 (10)	8.2 (25)	9.1 (19)	11.3 (25)	13.1 (24)	<.0001
**Married/Nonmarital union**^3^	85.2 (178)	84.6 (197)	83.7 (206)	80.6 (214)	82.8 (212)	0.2695	77.1 (266)	66.4 (202)	60.0 (126)	65.8 (144)	56.9 (103)	<.0001
**City resident**	62.2 (130)	68.2 (159)	64.5 (158)	64.3 (173)	72.0 (190)	0.1075	76.5 (264)	77.0 (234)	82.8 (173)	76.1 (169)	76.4 (139)	0.9424
**Previous livebirth**	20.6 (43)	26.3 (61)	26.4 (65)	30.9 (83)	37.9 (100)	<.0001	26.7 (92)	42.8 (130)	43.3 (91)	42.8 (95)	41.5 (76)	0.0003

**Perinatal characteristics**												
**Unintended pregnancy**	52.2 (109)	34.2 (79)	25.4 (62)	27.2 (67)	30.2 (70)	<.0001	65.8 (225)	63.8 (192)	55.1 (109)	52.2 (105)	46.0 (63)	<.0001
**Start of prenatal care**												
≤28 weeks	77.5 (162)	82.8 (193)	85.4 (210)	84.8 (228)	83.7 (221)	0.0826	60.9 (210)	51.3 (156)	55.7 (117)	66.2 (147)	66.7 (122)	0.0214
>28 weeks	4.8 (10)	3.9 (9)	4.9 (12)	2.6 (7)	4.2 (11)	0.5403	6.4 (22)	6.3 (19)	4.3 (9)	3.2 (7)	3.8 (7)	0.0508
None	17.7 (37)	13.3 (31)	9.8 (24)	12.6 (34)	12.1 (32)	0.1136	32.8 (113)	42.4 (129)	40.0 (84)	30.6 (68)	29.5 (54)	0.1428
**Injection drug use during pregnancy**	N/A	N/A	N/A	N/A	N/A	N/A	51.6 (178)	59.5 (181)	61.9 (130)	56.8 (126)	62.3 (114)	0.0387
**Preterm delivery**	10.7 (22)	15.7 (36)	14.4 (35)	15.2 (40)	19.7 (51)	0.0199	31.8 (108)	36.0 (108)	34.8 (72)	31.7 (70)	38.1 (69)	0.4036
**Mode of delivery**												
Elective cesarean delivery	5.3 (11)	7.4 (17)	7.4 (18)	8.0 (21)	7.7 (20)	0.3419	2.3 (8)	4.0 (12)	3.9 (8)	4.5 (10)	5.5 (10)	0.0684
Emergency cesarean delivery	9.7 (20)	9.6 (22)	12.4 (30)	13.4 (35)	15.4 (40)	0.0233	11.1 (38)	6.3 (19)	4.8 (10)	9.1 (20)	15.5 (28)	0.2114
Vaginal	85.0 (175)	83.0 (190)	80.3 (195)	78.6 (206)	76.8 (199)	0.0124	86.6 (296)	89.7 (271)	91.3 (189)	86.4 (191)	79.0 (143)	0.0347

**Maternal clinical characteristics**												
**HIV Diagnosis at** **Labor/Delivery**	12.5 (26)	11.2 (26)	6.9 (17)	8.2 (22)	9.9 (26)	0.2089	18.6 (64)	26.0 (79)	22.4 (47)	21.2 (47)	18.6 (34)	0.8095
**Hepatitis C**	39.2 (82)	33.9 (79)	28.1 (69)	32.7 (88)	24.2 (64)	0.0013	89.3 (308)	90.5 (275)	96.2 (202)	88.7 (197)	88.0 (161)	0.7412
**CD4 Test Performed**	57.9 (121)	61.8 (144)	73.6 (181)	83.3 (224)	80.3 (212)	<.0001	47.5 (164)	39.1 (119)	42.4 (89)	55.0 (122)	63.9 (117)	<.0001
**CD4 count**												
≤200	0.8 (1)	2.8 (4)	2.8 (5)	3.1 (7)	2.8 (6)	0.3366	3.1 (5)	5.0 (6)	6.7 (6)	9.0 (11)	8.6 (10)	0.0200
201-350	14.1 (17)	9.7 (14)	9.4 (17)	10.3 (23)	9.0 (19)	0.2599	12.8 (21)	8.4 (10)	13.5 (12)	8.2 (10)	12.8 (15)	0.8713
>350	85.1 (103)	87.5 (126)	87.9 (159)	86.6 (194)	88.2 (187)	0.5740	84.2 (138)	86.6 (103)	79.8 (71)	82.8 (101)	78.6 (92)	0.1771
**Viral Load Test Performed**	10.0 (21)	18.1 (42)	33.7 (83)	54.7 (147)	75.4 (199)	<.0001	7.5 (26)	7.9 (24)	20.5 (43)	32.4 (72)	57.9 (106)	<.0001
**Viral load**												
>10,000	42.9 (9)	23.8 (10)	22.9 (19)	18.4 (27)	8.5 (17)	<.0001	42.3 (11)	54.2 (13)	23.3 (10)	22.2 (16)	12.3 (13)	<.0001
1,001-10,000	38.1 (8)	42.9 (18)	36.1 (30)	26.5 (39)	10.1 (20)	<.0001	34.6 (9)	29.2 (7)	32.6 (14)	34.7 (25)	16.7 (18)	0.0362
≤1,000	19.1 (4)	33.3 (14)	41.0 (34)	55.1 (81)	81.4 (162)	<.0001	23.1 (6)	16.7 (4)	44.2 (19)	43.1 (31)	70.8 (75)	<.0001

**Prevention of mother-to-child transmission and infant abandonment**												
**Start of prenatal antiretroviral therapy**												
≤28 weeks	44.9 (92)	56.1 (129)	67.1 (161)	72.6 (188)	72.3 (185)	<.0001	44.4 (152)	35.9 (107)	40.3 (81)	50.7 (109)	54.2 (96)	0.0032
>28 weeks	34.2 (70)	24.4 (56)	18.8 (45)	10.8 (28)	9.8 (25)	<.0001	19.0 (65)	17.5 (52)	12.4 (25)	7.9 (17)	7.9 (14)	<.0001
None	21.0 (43)	19.6 (45)	14.2 (34)	16.6 (43)	18.0 (46)	0.2958	36.6 (125)	46.6 (139)	47.3 (95)	41.4 (89)	37.9 (67)	0.7900
**Type of antiretroviral prophylaxis**												
Full course dual/triple ARV prophylaxis^4^	13.9 (29)	15.0 (35)	18.7 (46)	55.8 (150)	59.1 (156)	<.0001	9.3 (32)	5.3 (16)	17.6 (37)	41.9 (93)	43.7 (80)	<.0001
Full course AZT prophylaxis^5^	61.2 (128)	59.7 (139)	64.2 (158)	22.7 (61)	20.5 (54)	<.0001	50.4 (174)	45.1 (137)	33.3 (70)	16.2 (36)	16.9 (31)	<.0001
Incomplete ARV prophylaxis^6^	24.9 (52)	25.3 (59)	17.1 (42)	21.6 (58)	20.5 (54)	0.1534	40.3 (139)	49.7 (151)	49.1 (103)	41.9 (93)	39.3 (72)	0.6076
**Infant follow-up**	75.5 (154/204)	69.6 (156/224)	71.7 (172/240)	66.5 (173/260)	41.8 (107/256)	<.0001	84.7 (282/333)	78.9 (232/294)	82.8 (168/203)	66.1 (142/215)	32.8 (58/177)	<.0001
**Perinatal HIV transmission^7^**	3.9 (6/154)	5.8 (9/156)	3.5 (6/172)	2.9 (5/173)	NA	0.4250	6.0 (17/282)	10.3 (24/232)	7.1 (12/168)	3.5 (5/142)	NA	0.3851
**Infant abandonment**	5.5 (11/202)	3.2 (7/221)	3.0 (7/237)	4.3 (11/257)	3.5 (9/255)	0.5531	15.6 (51/326)	20.2 (55/273)	18.8 (33/176)	9.8 (20/205)	9.9 (17/172)	0.0090

Petersburg, Russia, 2004-2008, enhanced perinatal HIV surveillance.

^1^N varies due to missing data.

^2^*P*-value from Cochran-Armitage two-sided test.

^3^Compared with never married, widowed/divorced or unknown

^4^Dual (typically AZT + 3TC) or triple (typically AZT+3TC+LPV/r) ARV prenatally, intravenous AZT intranatally and AZT syrup neonatally

^5^AZT monotherapy prenatally, intravenous AZT intranatally and AZT syrup neonatally

^6^Single-doze NVP intranatally and/or NVP syrup (or incomplete course of AZT syrup) neonatally

^7^Delayed establishment of infant HIV status for many infants born in 2008 did not allow calculation of perinatal HIV transmission.

During the study period, we observed several significant changes in the characteristics of HIV-infected women giving birth. The proportion of women aged >30 years increased regardless of injection drug use history (2.9%-17.4% among non-IDUs, 2.9%-13.1% among IDUs); similarly and potentially related to this age trend, the proportion of women who had previous live births increased (20.6%-37.9% among non-IDUs, 26.7%-41.5% among IDUs) (all *P *< 0.001, Table 1). The majority of women giving birth were married or living in nonmarital unions and were city residents, and thus eligible for health-related and social services provided by the city (other HIV-infected women who live in the city but don't have official registration are usually eligible only for emergency services, including delivery, but not for routine HIV treatment, care and support). We observed a decreasing trend of unintended pregnancy (52.2%-30.2% among non-IDUs, 65.8%-46.0% among IDUs, both *P *< 0.0001). The proportion of women without prenatal care remained relatively stable, ranging from 9.8%-17.7% among non-IDUs, and from 29.5%-42.4% among IDUs. Among women who did receive prenatal care, we observed the tendency of its earlier initiation: e.g., the proportion of HIV-infected women who started prenatal care ≤28 weeks of gestation increased from 60.9% to 66.7% (*P *< 0.05) among IDUs. Although the proportion of preterm deliveries was lower among non-IDUs, it almost doubled in this group of women (10.7%-19.7%, *P *< 0.05). The percentage of women with vaginal deliveries decreased (85.0%-76.8% among non-IDUs, 86.6%-79.0% among IDUs, both *P *< 0.05).

**Figure 3:**
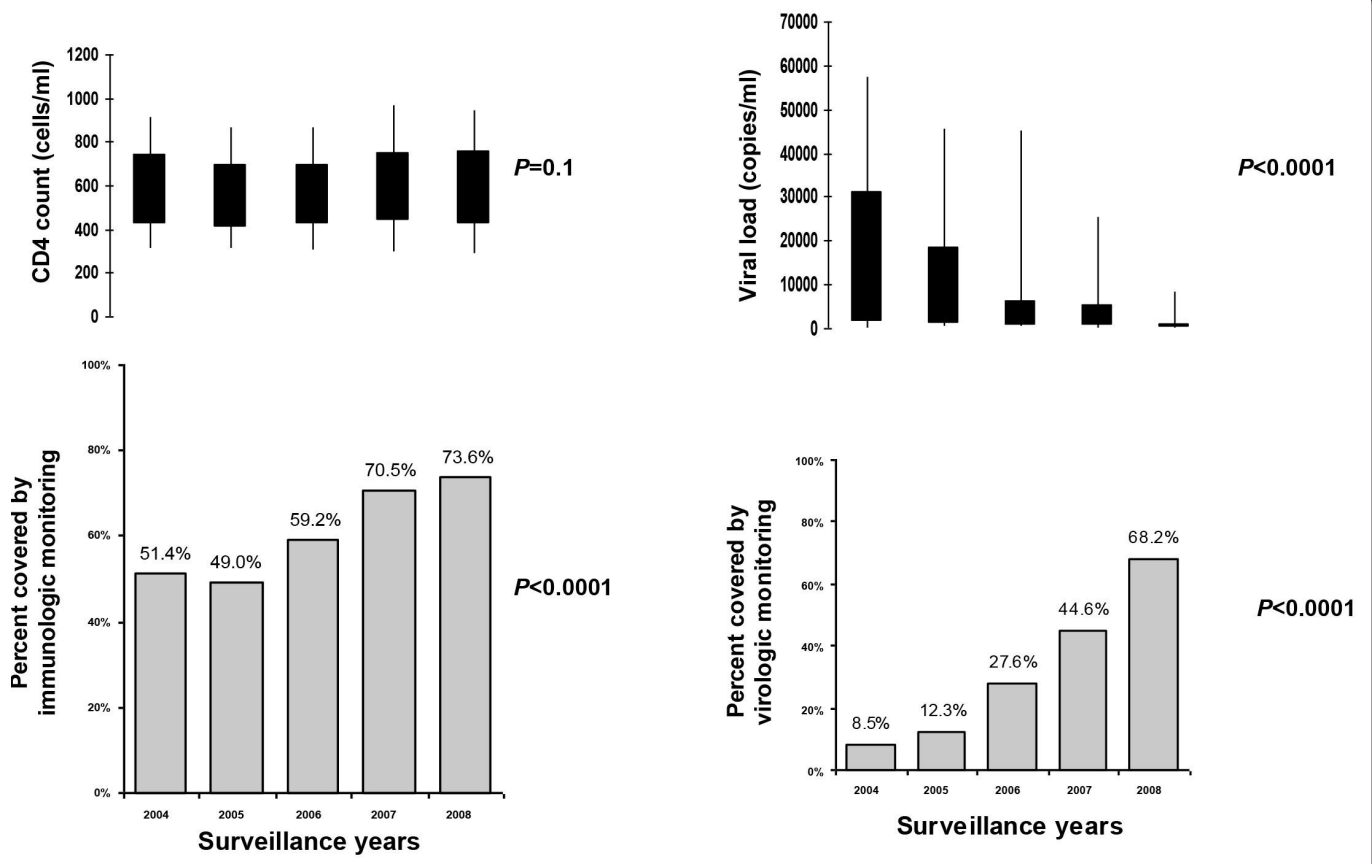
**CD4-cell count and viral load testing among HIV-infected women giving birth**. CD4-cell counts and percentage covered by immunologic monitoring, viral load and percentage covered by virologic monitoring, HIV-infected women giving birth, St. Petersburg, Russia, 2004-2008, enhanced perinatal HIV surveillance.

Uptake of immunologic and virologic monitoring in both non-IDUs and IDUs also increased (Table 1). Overall, in non-IDUs and IDUs combined, the proportion of women who received CD4-cell count testing and viral load testing increased significantly (51.4%-73.6% and 8.5%-68.2%, respectively, both *P *< 0.0001, Figure 3). Median CD4-cell count remained relatively stable at 565 cells/mL (IQR: 422-732 cells/mL), and median viral load (community viral load) decreased from 9,170 copies/mL (IQR: 1,420-31,100 copies/mL) to 201 copies/mL (IQR: 60-794 copies/mL) (*P *< 0.0001).

**Figure 4:**
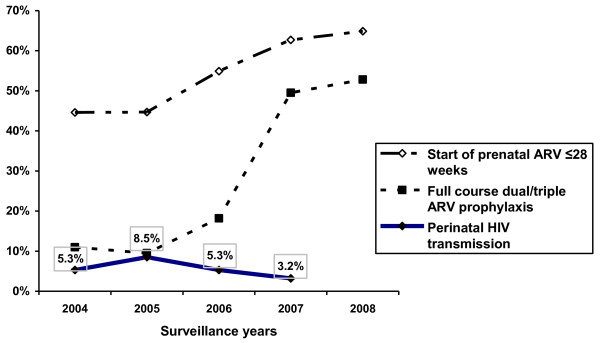
**Timing and completeness of antiretroviral prophylaxis and perinatal HIV transmission**. Percentage of HIV-infected women who started prenatal ARV ≤28 weeks gestation and received a full course dual/triple ARV prophylaxis, rate of perinatal HIV transmission, St. Petersburg, Russia, 2004-2008, enhanced perinatal HIV surveillance.

Significant changes in the timing and completeness of antiretroviral prophylaxis during the five years of surveillance occurred among both non-IDUs and IDUs (Table 1). For the two groups combined, both initiation of antiretroviral prophylaxis ≤28 weeks gestation and dual/triple antiretroviral prophylaxis increased (44.6%-64.9% and 11.0%-52.8%, respectively, both *P *< 0.0001, Figure 4). Overall, infant follow-up was over 60% for the first four years of surveillance (2004-2007) (Table 1). It is important to note that since there is continuous work to increase follow-up of HIV-exposed infants who do not have determined HIV status, the rate of infant follow-up for a given year represents outreach activities conducted during the subsequent years.

The overall rate of perinatal HIV transmission during the 4-year surveillance period was 4.0% (CI 2.7%-5.8%) and 7.0% (CI 5.5%-9.0%) among non-IDUs and IDUs, respectively. For all HIV-infected women giving birth in St. Petersburg, perinatal HIV transmission initially increased from 5.3% (CI 3.5%-7.8%) in 2004 to 8.5% (CI 6.1%-11.7%) in 2005 (*P *< 0.05), followed by a decline to 5.3% (CI 3.4%-8.3%) in 2006, and 3.2% (CI 1.7%-5.8%) in 2007 (*P *for trend <0.05, Figure 4). The rates of infant abandonment decreased significantly from >15% during the first three years of surveillance (2004-2006) to <10% during 2007-2008 (*P *< 0.01) among IDUs, and remained unchanged at 3.0%-5.5% among non-IDUs (Table 1).

It is noteworthy that for most of the study years considered, IDUs were more likely than non-IDUs to be unmarried, to have unintended pregnancy, later initiation of or no prenatal care, preterm delivery, to receive HIV diagnosis in labor and delivery, to have hepatitis C, no immunologic and virologic monitoring, lower CD4 count, later or incomplete antiretroviral prophylaxis, higher rates of perinatal transmission and infant abandonment (Table 1).

## Discussion

These analyses of five years of data from standard and enhanced perinatal HIV surveillance provide important information about trends in the epidemiology and prevention of mother-to-child HIV transmission in St. Petersburg-one of the cities in Russia most affected by HIV. We observed a significant decrease in births among all HIV-infected women, and a decrease in IDUs among HIV-infected women giving birth. Regardless of injection drug use history, we noted impressive improvements in the uptake of clinical services by HIV-infected women giving birth, such as earlier initiation of prenatal care, fewer unintended pregnancies, higher uptake of immunologic and virologic monitoring, earlier initiation and more complete antiretroviral prophylaxis. Most significantly, these improvements in clinical services were followed by a decrease in the rates of mother-to-child HIV transmission from 2005 to 2007.

Our data show that in contrast to the increasing birth rate in the general population of women in St. Petersburg, HIV-infected women are having fewer births. The declining birth rate among HIV-infected women may be a result of increasing age of these women or indicate a decrease in their fertility desires, and/or improved family planning because no increase of abortions was observed in this population during the same time period (personal communication with Dr. Nikolay Belyakov, Director, St. Petersburg City AIDS Center, where 90% of abortions among HIV-infected women are performed). During the last two years of assessment, many HIV-infected women of reproductive age in St. Petersburg received free family planning services, including the contraceptives of their choice [16]. These programmatic improvements likely contributed to the decreases in the birth and unintended pregnancy rates among HIV-infected women. In addition, we observed a decreasing proportion of IDUs among HIV-infected women giving birth, which might be explained by ongoing heterosexual transmission of HIV from HIV-infected male IDUs and some heterosexual transmission outside of the traditional high-risk groups. The improvements of clinical HIV services, albeit more pronounced among women without a history of injection drug use, were evident for both non-IDUs and IDUs.

Perinatal HIV transmission during the first year of surveillance increased, coinciding with no improvements in timing or completeness of antiretroviral prophylaxis. The subsequent decrease of perinatal transmission was most likely due to multiple factors, although earlier initiation and higher effectiveness of antiretroviral prophylaxis were probably the main contributors [9]. Other factors that may have contributed to the decrease in perinatal HIV transmission include a lower rate of unintended pregnancies, earlier initiation of prenatal care, and improved immunologic and virologic HIV monitoring. However, not all women had the benefits of improved clinical services. The steady proportion of women who did not receive prenatal care and ARV prophylaxis, and the increasing trend of injection drug use during pregnancy among IDUs, both indicate that outreach programs are not reaching all women.

Standard HIV surveillance in pregnant women provides important data on trends of the epidemic and overall effectiveness of preventive measures [17], yet contributes minimally to identifying specific areas for programmatic improvement. Enhanced perinatal surveillance, on the other hand, provides supplementary detailed information on the mother-infant pair, including risk factors, clinical services, and laboratory data, which assists timely evaluation of perinatal prevention efforts [18,19]. It can be linked with other systems or registries, such as, for example, maternal and infant records at the City AIDS Center. The annual cost of enhanced perinatal surveillance in St. Petersburg was approximately $20,000. If scaled up to a total of five metropolitan areas with highest HIV seroprevalence (i.e., Samara, Irkutsk, Yekaterinburg, Orenburg) [6], enhanced perinatal surveillance may provide valuable national data on the risk factors for perinatal HIV transmission for considerably less than one percent of the amount spent on HIV prevention in Russia [20]. In St. Petersburg, enhanced perinatal surveillance was critical in identifying areas needing improvement, such as limited use of effective family planning [21], low infant follow-up [10], and delayed and less effective antiretroviral prophylaxis [9]. Immediate, focused attention by the local public health leadership made it possible to address each of these issues in a timely fashion through program and policy improvements. As a result, we observed fewer unintended pregnancies, improved infant follow-up, earlier and more effective antiretroviral prophylaxis, and, subsequently, fewer HIV-infected infants.

Our assessment supports the evidence from other countries-improvement of clinical services for HIV-infected women results in significant reductions in perinatal HIV transmission. The success of high-income countries in reducing perinatal HIV transmission was attributed to increased coverage of HIV-infected pregnant women by combination antiretroviral prophylaxis, elective cesarean delivery, and avoidance of breastfeeding [22-24]. Despite a number of challenges in low- and middle-income countries [25,26], a few reports provide evidence that it is possible to reduce perinatal HIV transmission with implementation of comparable preventive measures [27,28]. In Ukraine, which had a healthcare system similar to the one in Russia, but had lower coverage by combination antiretroviral prophylaxis at the time of assessment, perinatal HIV transmission was reduced in half in five years (from 15.2% in 2001 to 7.0% in 2006) by strengthening clinical services provided to HIV-infected women [27]. In Russia, where the rate of perinatal HIV transmission has been relatively stable at 6%-8%, the St. Petersburg experience suggests that it is feasible to attain low rates of mother-to-child transmission similar to U.S. and Western European rates. Since our observation of the impact of early and effective antiretroviral prophylaxis on perinatal HIV transmission is consistent with findings from randomized controlled trials from various parts of the world [29], the data described in this report may be generalizable to other middle-income countries with a large proportion of hospital deliveries and replacement feeding among HIV-infected women giving birth.

The results of this assessment should be interpreted in light of its strengths and limitations. Strengths of the study include almost universal coverage of HIV-infected women giving birth by enhanced perinatal surveillance and standardized data collection methods that allow trend analyses. Although ours is one of the few reports to describe trends in critical indicators of perinatal HIV transmission separately for non-IDUs and IDUs, it is possible that due to social desirability, some women with a history of injection drug use were misclassified as non-IDUs. In additional subgroup analysis (not shown), critical indicators of perinatal transmission among women who reported no history of injection drug use but had hepatitis C coinfection more closely resembled non-IDUs, indicating that the effect of any misclassification of self-reported injection drug use was likely to have been minimal. Another limitation of the study is a large proportion of HIV-exposed infants with undetermined HIV status (e.g., 33.7% during 2007). Previous analysis showed that characteristics of women whose infants had known HIV status were similar to that of the entire population of HIV-infected women. There were, however, some differences: infant HIV status was more likely to be unknown for subgroups with both increased (nonresidents and those with late initiation of antiretroviral prophylaxis) and decreased (those using injection drugs during pregnancy) risk factors for transmission [9]. Therefore, HIV transmission rates can be either underestimated or overestimated.

### Conclusion

To our knowledge, this report is the first to document a successful reduction in perinatal HIV transmission in one of the most affected regions in Russia. Our report provides evidence that targeted and comprehensive HIV prevention measures are effective. In addition, this report demonstrates the vital role of enhanced perinatal surveillance in driving programmatic improvements. Scaling up enhanced perinatal surveillance in other key Russian regions will allow effective improvement in local perinatal HIV programs and provide useful data to monitor trends in perinatal HIV transmission. Our data also suggest strategies to further reduce perinatal transmission, which include widespread opt-out HIV testing of women before and during pregnancy and increased outreach to high-risk HIV-infected women to avoid consequences of drug use and facilitate their early contact with and retention in the healthcare system when they become pregnant or are planning pregnancy. In addition, further reductions in perinatal HIV transmission will require full access to effective and affordable family planning services for HIV-infected women. We believe that Russia has the tools to successfully prevent perinatal HIV. The availability of effective antiretroviral regimens, an adequate infrastructure for elective cesarean delivery, and the possibility to safely avoid breastfeeding, should make it feasible to reduce and even eliminate perinatal HIV transmission in Russia.

## Competing interests

The authors declare that they have no competing interests.

## Authors' contributions

All authors contributed to study conception and design; NA, NAB, AGR, EEV, GVV, AAY contributed to local study implementation; MGM, DMK, SH, DJJ and CV contributed to data analysis and/or interpretation; DMK drafted the manuscript; all authors reviewed, critically revised, and approved the manuscript.

## Pre-publication history

The pre-publication history for this paper can be accessed here:

http://www.biomedcentral.com/1471-2334/11/292/prepub
